# Managing a patient with open-angle glaucoma: a case study

**Published:** 2012

**Authors:** Fatima Kyari, Mohammed M Abdull

**Affiliations:** Ophthalmologist: Department of Ophthalmology, College of Health Sciences, University of Abuja, Nigeria.; Ophthalmologist: Ophthalmology: Department, Abubakar Tafawa Balewa University Teaching Hospital, Bauchi, Nigeria.

**Figure:**
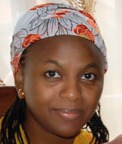
Fatima Kyari

**Figure:**
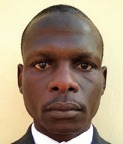
Mohammed M Abdull

**PANEL OF EXPERT GLAUCOMA SPECIALISTS**

**Figure:**
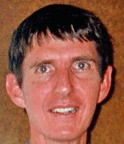
**Colin Cook** Professor of Ophthalmology, University of Cape Town; CBM Eye Medical Advisor.

**Figure:**
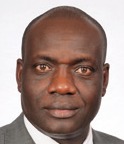
**Dan Kiage** Head of Ophthalmology, Aga Khan University Hospital, Kenya.

**Figure:**
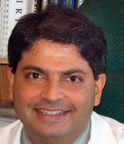
**Karim F Damji** Professor of Ophthalmology, University of Alberta.

**Figure:**
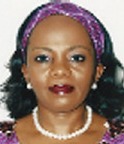
**Adeola Onakoya** Consultant Ophthalmologist, College of Medicine, Lagos University Teaching Hospital, Lagos, Nigeria.

## Case presentation

**Figure:**
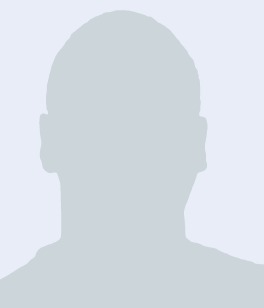


Mr AA is a 48-year-old shop attendant who presented at the eye unit of a teaching hospital with a history of gradual, painless vision loss. His presenting (unaided) visual acuity was counting fingers at 1 metre in the right eye and 6/60 in the left eye. Both corneas were clear, and the pupils had a slow reaction to light. There was a right relative afferent pupillary defect (RAPD). The right eye had a nuclear sclerotic cataract which precluded a good view of the optic nerve head, and a vertical cup:disc ratio (VCDR) of about 0.9, barely visible through the dilated pupil with the binocular indirect ophthalmoscope. The left eye VCDR was 0.8. Intraocular pressure (IOP) was 32 mmHg (right eye) and 30 mmHg (left eye) by applanation tonometry. Gonioscopy showed open angles in both eyes. Visual field tests (standard automated perimetry [SAP]) could not be carried out.

## How would the panel manage Mr AA?

Most of the panellists mentioned the importance of talking to Mr AA about glaucoma and what his treatment options were. Some mentioned asking a nurse counsellor to talk to the patient.

The next important issue to be addressed was the setting of a target IOP in the lower teens, and discussing this target with the patient.

There was general agreement that the initial control of IOP should be by medical treatment, while preparing for surgery on the right eye. First choice was a combination of a beta-blocker and a prostaglandin analogue (PGA). A second option was a combination of a beta-blocker and an alpha-agonist. The panel mentioned the need to bear in mind the cost and availability of the drugs.

All panellists agreed that the right eye should be treated first, and firmly recommended a combined procedure: cataract with posterior chamber intraocular lens (PCIOL), and trabeculectomy with adjunctive antimetabolite therapy. The reasons were both clinical and patient related:

“A trabeculectomy alone may give better IOP control, but will likely worsen vision and, depending on the techniques available and how the bleb turns out, going back to take out the cataract could create inflammation and/or directly compromise the bleb and worsen IOP control.”

“Cataract surgery alone is out of the picture, since a serious IOP spike could wipe out remaining visual field and adequate IOP control is not likely to be achieved.”

“The patient will better understand the benefit of surgery [and therefore be more likely to attend further appointments] if he can be offered some visual improvement.”

Depending on the centre and available facilities, the suggested approaches for surgery on the right eye were:

- phacoemulsification with PCIOL and trabeculectomy
- small incision cataract surgery (SICS) with PCIOL and trabeculectomy at a separate site
- extra-capsular cataract extraction (ECCE) with PCIOL and trabeculectomy.

Adjunct therapy could be with:

- beta irradiation applied with a strontium plaque
- mitomycin C (MMC)
- 5-fluorouracil (5FU).

**Figure:**
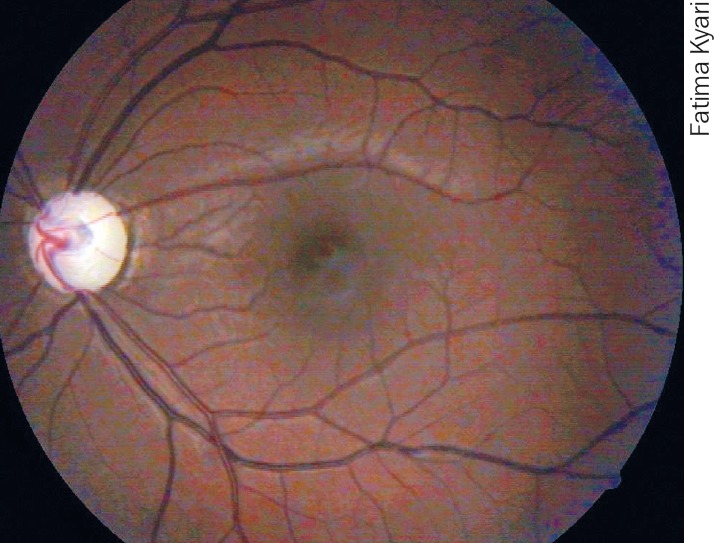
End-stage glaucoma: disc-cupping

Adjunct therapy is to prevent bleb scarring, however, there is little evidence that MMC or 5FU make any difference in combined procedures.

There is some evidence to support using separate sites rather than the same site in combined phacoemulsification and trabeculectomy surgery.

The choice of treatment for the left eye was not so uniform across the panel. Having initiated medical treatment for IOP control, a top choice was to perform a trabeculectomy with adjunct 5FU or MMC. However, some panellists said they would only offer surgery if there was inadequate IOP control with medications; others would also offer laser treatment as an option.

Both eyes would also have refraction, and the patient would be given spectacles if needed.

## Additional comments from panelists

“Patients are becoming more informed and are likely to seek more information and ask for more choices, regardless of their literacy or socioeconomic levels. Therefore, counselling needs to be more comprehensive, to include the biological situation of the eye and whole body, the patient's psychological perceptions, their social and economic situation, as well as their religious beliefs.”

“The role of counsellors cannot be overemphasised, as they will take more time to explain to the patient the pros and cons of staying away or declining surgery.”

“The nurse counsellor could keep a register with the patient's mobile phone number. She could sms (text) or phone him if he defaults on follow-up.”

“When the mode of treatment is certain and options are limited, like in the case of the right eye, then be firm to recommend that to the patient.”

“In the absence of a visual field test machine, assessment can be done very simply, by confrontation visual field testing, with a red pin or fingers [see page 68]. Some people have abnormally large discs, which may seem to indicate pathology, but have normal visual fields.”

“If it is not possible to visualise the disc, for example because of cataract, be guided by the patient's IOP and by the results of visual field tests, however basic.”

“It is important to carefully assess for RAPD because the disease is asymmetric. In the absence of any other formal function test (such as visual fields) RAPD is a very useful clinical sign in glaucoma, because it provides objective evidence of functional loss [see page 58].”

## Full case and management

After the panelists outlined their management plan for Mr AA, they were given the full case and details of the management that was actually undertaken in his presenting hospital.

Mr AA was diagnosed with glaucoma and cataract at his initial presentation. At that time he was told he had advanced eye disease and needed to have surgery to preserve his vision. He asked whether the operation would make him see better. He was frankly informed that it would only preserve the vision he had at that time in the left eye; and that, if the cataract was causing much of the poor vision in the right eye, his vision in that eye would improve after cataract surgery.

Medical treatment with eye drops (xalatan and timolol) was recommended, and Mr AA was given one month to make a decision about surgery. He was told to get the prescribed medications in the meantime and to start using them.

Mr AA did not return until six months later. He said that he had bought one bottle each of the eye drops, but could not buy more because they were expensive. He decided not to come back to the clinic because he was sure the doctor would be angry with him. At that stage he decided to see a traditional healer on the recommendation of a close family friend.

When this did not work, Mr AA went to a different eye clinic near his home where he was told he had cataract and needed to go to hospital for surgery. This brought him back to the same eye unit, where visual field assessment by confrontation was attempted.

This showed substantial loss of his peripheral visual field: Mr AA was only able to see fingers when they were presented in the centre of his visual axis.

Mr AA was informed that his vision had deteriorated further since the last time he was seen, and that if this continued he would lose vision permanently in both eyes. He was offered combined cataract surgery and trabeculectomy in the right eye, and trabeculectomy only in the left eye. The right eye would be operated on first.

Surgery, rather than medical treatment, was offered because it was clear from past experience that he would not be able to afford to use the more effective eye drops on a regular basis: surgery would be a one-time procedure which would be cheaper for him in the long run.

**‘The uptake of glaucoma surgery still seems very low in Africa’**

The decision to offer combined trabeculectomy and cataract surgery was made based on the patient's record of defaulting on follow-up. Removal of the cataract from the right eye would provide him with some improvement in vision as well as IOP control, which would hopefully motivate him to present for trabeculectomy in the left eye at a later time.

Mr AA agreed that he would have the operation this time, but said he wanted time to talk to his family about how they could make the money available. As he could not afford xalatan, he was then asked to use only timolol until the surgery date. Pilocarpine, even though less costly, was not an option for him as cataract surgery was being planned.

Mr AA was given two weeks to make a decision and return.

He returned after three weeks, explaining that the person accompanying him had been away. However, he came prepared to have surgery and was admitted for surgery immediately so as not to lose him.

The standard surgery usually offered at the hospital is manual small-incision sutureless cataract surgery. Mr AA was initially offered right ECCE and PCIOL, because combined SICS and trabeculectomy can be more difficult to perform. However, the final decision was to offer SICS with PCIOL at a temporal site, and simultaneous trabeculectomy with MMC at a more nasal position. The decision to use MMC was to prevent bleb scarring.

Mr AA's immediate post-operative unaided visual acuity in the operated eye was 4/60. He was also informed about the importance of adherence to prescribed medication and follow-up after the operation.

Mr AA returned for his 1-month follow-up appointment and had a postoperative review of the right eye. His unaided visual acuity was 6/60; the bleb was draining and was not cystic; the IOP was 12 mmHg and he was pleased with his improved visual function.

There was some discussion about what to do about the left eye and he was asked to bring his first-degree relatives to the next appointment, so that they could be screened for glaucoma.

Mr AA underwent refraction of the left eye and had a corrected visual acuity of 6/18. IOP was controlled with timolol and xalatan (which Mr AA was able to buy using some of the funds he had set aside for the operation). However, because he expressed concern about not being able to afford life-long medication, left eye trabeculectomy with MMC was subsequently performed.

We are grateful to our reviewers, Clare Gilbert, Richard Wormald, and Nick Astbury for their contributions.

**Final comments by the panellists**

“An interesting case and very real in our setting. Mr AA highlights the problem that we all experience: non-compliance with topical medication and failure to return for regular follow-up.”

“The ophthalmologist made very reasonable decisions in the light of the prevailing circumstances.”

“Even challenging situations can lead to success, as seen in this case, at least in the short term.”

“Surgery is definitely the right approach in the management of this patient; otherwise the next time he returns his visual acuity may be further reduced.”

“The uptake of glaucoma surgery still seems very low in Africa. However, we should realise that, for many of our patients, surgery should be the first line of treatment. Nevertheless, there will still be patients who would adamantly refuse surgery, and for whom we would need to consider laser treatment, if available.”

“This case underscores the role of advocacy for universal health care to cover potentially blinding conditions such as glaucoma, as well as the need for greater public education and awareness. These are issues which the ophthalmologist cannot handle alone but which require engagement with government and other community development sectors.”

